# Reliable and Standardized Animal Models to Study the Pathogenesis of Bluetongue and Schmallenberg Viruses in Ruminant Natural Host Species with Special Emphasis on Placental Crossing

**DOI:** 10.3390/v11080753

**Published:** 2019-08-15

**Authors:** Ludovic Martinelle, Fabiana Dal Pozzo, Etienne Thiry, Kris De Clercq, Claude Saegerman

**Affiliations:** 1 CARE-FEPEX experimental station, Fundamental and Applied Research for Animals & Health (FARAH) Center, Faculty of Veterinary Medicine, University of Liege, 4000 Liege, Belgium; 2 Research Unit in Epidemiology and Risk Analysis Applied to Veterinary Sciences (UREAR-ULg), Fundamental and Applied Research for Animals & Health (FARAH) Center, Faculty of Veterinary Medicine, University of Liege, 4000 Liege, Belgium; 3 Veterinary Virology and Animal Viral Diseases, Fundamental and Applied Research for Animals & Health (FARAH) Center, Faculty of Veterinary Medicine, University of Liege, 4000 Liege, Belgium; 4 Infectious Diseases in Animals, Exotic and Particular Diseases, Sciensano, 1180 Brussels, Belgium

**Keywords:** Bluetongue, Schmallenberg, *Culicoides*, vector-borne disease, experimental challenge, infection, arboviruses

## Abstract

Starting in 2006, bluetongue virus serotype 8 (BTV8) was responsible for a major epizootic in Western and Northern Europe. The magnitude and spread of the disease were surprisingly high and the control of BTV improved significantly with the marketing of BTV8 inactivated vaccines in 2008. During late summer of 2011, a first cluster of reduced milk yield, fever, and diarrhoea was reported in the Netherlands. Congenital malformations appeared in March 2012 and Schmallenberg virus (SBV) was identified, becoming one of the very few orthobunyaviruses distributed in Europe. At the start of both epizootics, little was known about the pathogenesis and epidemiology of these viruses in the European context and most assumptions were extrapolated based on other related viruses and/or other regions of the World. Standardized and repeatable models potentially mimicking clinical signs observed in the field are required to study the pathogenesis of these infections, and to clarify their ability to cross the placental barrier. This review presents some of the latest experimental designs for infectious disease challenges with BTV or SBV. Infectious doses, routes of infection, inoculum preparation, and origin are discussed. Particular emphasis is given to the placental crossing associated with these two viruses.

## 1. Introduction

Amongst pathogens, RNA viruses were a major source of emerging diseases during the last 30 years [1]. High mutation rate and in case of segmented genome, reassortment are responsible for genetic adaptability and variability of these viruses.

Two pathogens affecting cattle and sheep were responsible for major outbreaks in Mainland Europe in the past 15 years: Bluetongue virus (BTV) and Schmallenberg virus (SBV). These outbreaks were singular in several ways: the diseases were previously either never reported in such northern locations (bluetongue virus) or recently discovered (Schmallenberg virus); their emergence still has unexplained aspects; both viruses displayed the ability to cross the placental barrier. Moreover, these events confirmed that palearctic endemic *Culicoides* species contribute to the spread of BTV and SBV and to the epizootic aspect of the diseases.

Bluetongue virus causes the eponymous bluetongue disease (BT). BTV belongs to the family *Reoviridae*, subfamily *Sedoreovirinae*, and represents the type specie of the *Orbivirus* genus [2]. The family *Reoviridae* currently contains fifteen genera of multi-segmented dsRNA viruses, including pathogens of a wide range of vertebrates (including humans), arthropods, plants, and fungi [3]. Unlike the other reoviruses, all orbiviruses are arthropod-borne viruses (arboviruses). This genus currently contains 22 species as well as 10 unclassified “orbiviruses” [4].

Until recent nomenclature changes implemented by the International Committee on Taxonomy of Viruses [5] Schmallenberg virus was part of the *Bunyaviridae* family, genus *Orthobunyavirus*, grouped within the serogroup Simbu along with at least 27 other virus species. The members of the Simbu serogroup show cross-reactions to the complement fixation test but are distinguished by seroneutralization [6] and by genetic sequence analysis. Yet still part of the *Orthobunyavirus* genus, SBV, AKAV and Aino virus (AINOV) are now considered exemplar viruses of the species *Sathuperi orthobunyavirus,* Akabane *orthobunyavirus*, and *Shuni orthobunyavirus*, respectively [7]. These belong to the new order *Bunyavirales,* family *Peribunyaviridae* (formerly *Bunyaviridae*)*,* which comprises the genus *Orthobunyavirus* and *Herbevirus* (host range limited to insects).

Despite their belonging to different viral families, BTV and SBV have several features in common. These converging aspects warrant the present work discussing more specifically the elements to consider while designing experimental infections targeting ruminant host species. A particular emphasis will be given to placental crossing and teratogenic potential of these two viruses.

## 2. Studying the Pathogenesis and Immune Response of BTV and SBV in Natural Ruminant Host Species

Experimental infections of mammalian hosts proved to be a highly valuable tool to study pathogenicity, virulence, pathogenesis, and transplacental infections since the dawn of the study of infectious diseases [8]. Design of in vivo models evolved and were usefully complemented with in vitro and in silico approaches to better comprehend the host-pathogen interactions.

Prior to study the pathogenesis of BTV and SBV in ruminants, including teratogenic potential, experimental models reproducing the disease had to be found.

To date there are no lab-adapted colonies of Palearctic *Culicoides*. Given the feeding behaviour of *Culicoides*, investigating the most adapted route of inoculation is of prime importance to ensure standardization and repeatability of challenge experiments. Amongst other important pathogenesis factors to consider when designing experimental infections, the origin of the inoculum and its passage history has to be carefully evaluated. Indeed the number of passages, the cell culture system used to grow the inoculum or by contrast, infectious blood or serum source are central to achieve experimental infection matching virological, clinical, and serological parameters of field infection with wild-type viruses.

### 2.1. Selection of an Appropriate Inoculum is Crucial to Achieve Adequate Experimental Infection 

Adequate inoculum to use in infectious challenges in order to study viral pathogenesis should be:

(1) Safe, meaning it should have been screened for contaminations, adventitial agents or other pathogens [9];
(2) Easily available, practical and standardised; and
(3) Made of a virus displaying similar replication and virulence properties than wild-type.

#### 2.1.1. Infectious Blood versus Cell Passaged Inoculum

A high quality infectious inoculum reproducing the pathogenesis of diseases was essential to investigate vaccine efficacy requirements or certain specific aspects of the pathogenesis of recently discovered viruses [10,11]. Facing an emerging disease with epizootic potential, reproduction of clinical disease under experimental conditions might be more reliable using infectious animal products such as blood or serum. Nevertheless, it appears that in the majority of the most recent experimental infections involving BTV or SBV, cell culture grown inocula were preferred for challenges (Table 1). Reasons to use cell-passaged viruses can be summarized as follow:

(1) Original isolate or any strain of particular interest can be shipped almost anywhere in the world, leading to great improvement of standardization;
(2) Viral amplification by cell-passages allows a high increase in viral titre, subsequently allowing to inoculate lower volumes;
(3) Screening for contamination or other pathogens is easier in cell culture and eliminate some veterinary public health concerns about using ruminant blood to infect other ruminants; and
(4) Virulence in cell culture can be easily standardized.

In several recent studies [14] clinical signs reported in BTV infected animals were of a lesser extent than those reported from the field [14,17,27,28,32,33,34]. As modified live vaccines gain their attenuation through serial cell passages, the first and most obvious hypothesis to explain the mild severity of bluetongue disease in the experiment is the use of culture grown virus. Moreover, passage history of the inocula used involved mostly embryonated chicken eggs (ECE), BHK-21 and VERO cells. It was reported that BTV grown on KC cells (derived from *Culicoides sonorensis*) could induce a greater clinical signs severity [35] probably because KC cells better mimic natural vector-borne infection compared to virus passaged on other cell lines [36].

Like other families of RNA viruses, *Peribunyaviridae*-RNA-dependent RNA polymerases and orbiviruses VP1 RNA-dependent RNA polymerase are prone to produce errors during viral genome replication. In general, RNA virus replication is characterized by high mutation rates (10^−5^–10^−3^ misincorporations per nucleotide copied), short generation times and high progeny yields [37]. In addition, segmented RNA viruses also generate genomic variations through recombination and reassortment [38]. Therefore, RNA viruses form populations of closely related viral variants that started from a single clone: the quasispecies [39,40].

With arthropod vectors, SBV and BTV typically undergo an alternate two-host ‘life cycle’ and are therefore suggested to be more stable and to evolve slower than vector-independent viruses [41]. Both steps may put selective pressures on these viruses, but it remains unknown whether sequence divergence is related to the mammalian or arthropod portion of life cycle. However, it has been demonstrated that despite the lack of changes in the consensus sequence, the passage of BTV in *Culicoides* cells induces an increase of the number of low-frequency variants as well as an increase in virulence [42]. This phenomenon has been hypothesized to explain the reduced viraemia and clinical picture seen in the re-emerging BTV8 in 2017 versus the 2007 strain [12].

As a matter of fact, it was also reported that the inoculation of infectious material from field isolates rarely produce a clinical picture as severe as in natural infection [19]. An additional hypothesis would be that *Culicoides* saliva might act as a catalyser enhancing the ability of BTV to produce severe clinical signs. Indeed *Culicoides* saliva was demonstrated to contain a trypsin-like protease able to cleave VP2, leading to infectious subviral particles formation with enhanced infectivity [43].

We demonstrated the suitability of BTV8 passaged a few times on cell culture to both reproduce clinical signs and RNA detection in calves [33]. Other authors concluded to the benefits of culture-grown viruses to be used in experimental challenges in ruminants [44], as well. Despite converging results, policy of the OIE remains unchanged regarding recommended vaccine efficacy requirements, i.e., challenging vaccinated and unvaccinated sheep with a virus “passaged only in ruminant animals and with no or limited ECE or cell culture passages” [45].

Results regarding SBV do not show that much consistency. In cattle, Wernike et al. reported a reduced viral replication of cell culture-grown SBV when compared to natural host-passaged inoculum [46]. By contrast, one year later the same team concluded to the suitability of both infectious serum and low passage cell culture material for SBV experimental challenges in sheep [47]. In addition to the passage history, the origin of the isolated virus seems to be of importance as virus originating from the central nervous system failed to reproduce RNAemia in inoculated animals [47]. Successive serial passages in cell-culture usually result in decreased virulence. However, regarding SBV, Varela et al. reported an increased pathogenicity in a SBV strain passaged 32 times in INF-incompetent sheep CPT-Tert cells, associated with a faster spread of the virus in the brain of suckling mice [48]. SBV was demonstrated to grow efficiently in several cell lines including sheep CPT-Tert, bovine BFAE, human 293T, dog MDCK, hamster BHK-21, BSR, KC, and VERO cells [48,49]. Whereas serial passages in CPT-Tert led to the accumulation of a variety of mutations mostly in the M and S segments, the porcine cell line SK-6 proved to be highly susceptible and to allow the genetic stability of SBV throughout successive passages [50]. Therefore, depending on the cell line used to grow SBV, serial passages can lead to attenuation, increased virulence, or efficient propagation with a low frequency of nucleotide exchanges.

#### 2.1.2. A Matter of Doses and Routes

When it comes to arboviruses the choice of the route of inoculation can be driven by two main considerations:

(1) The need for a route that best mimics the behaviour of the vector in field conditions. Usually haematophagous arthropods are either telmophagous or solenophagous; depending on the vector species the route might be intradermal (ID), subcutaneous (SC), or intravenous (IV). In experimental infections the inoculated viral load and volume are usually higher than the ones inoculated through naturally occurring feeding given the size of the arthropods and the size of their mouthparts [51]. Another drawback already mentioned is the lack of vector saliva components, which can modify the structure and infectivity of Reoviridae and Peribunyaviridae viral particles [43,52].
(2) The need for a route that will ensure the virus to reach the blood stream. Quite obviously this is the intravenous route. Since vector saliva components can enhance the infectivity of arboviruses there is a risk that the inoculation of the virus alone or at a distal site from the vector feeding site could result in a failed infection [53]. Therefore, the option to bypass the skin for reaching the bloodstream may be relevant.

Several authors including us [17] used mixed routes to overcome the respective disadvantages of each approach (Table 1; [34,54]). In a study of our group, we compared intranasal, intradermal and subcutaneous routes for experimental infections of ewes with SBV [28]. Intradermal is an interesting yet underused route: indeed most haematophagous arthropods do not pass the skin and their mouthparts only allow them to feed intradermally. Most of the cellular and fluid exchanges between the skin and the blood do occur in the dermis [55]. In addition, there are some evidences suggesting that intradermal inoculation can be more appropriate to reproduces many aspects of natural infection, including clinical disease, viral and immune responses [56]. The intradermic route was demonstrated to better mimic natural early stages of the infection, directly influencing the severity of the disease. BTV-induced immunosuppression is linked to the infection and disruption of follicular dendritic cells, which is mostly possible through intradermic inoculation [57].

However, to perform an actual intradermal inoculation the volume to be injected has to be limited, the dermis being mostly composed of a dense network of collagen fibres. Therefore, it is required to multiply inoculation sites to reach desired total inoculum volume and infectious titre. To realize the inoculation itself, the most practical tools are Dermojet^®^ (Akra Dermojet) or special syringes for intradermal injections (used to perform bovine tuberculosis skin tests for example). These devices allow usually volumes between 0.1 and 0.4 mL, thus the need for multiple injections to reach the common 1–4 mL inoculation volume used in ruminant infectious challenges experiments (Table 1). Moreover, with both systems the inoculum has to be transferred from its original vial to a small tank of the dermojet or to a special cartridge to be used with the intradermal syringe. This extra step increases the number of handlings, which should be limited especially in the case of BSL3 pathogens.

We investigated the intranasal route to test whether or not a potential direct SBV contamination between sheep could be achieved [28]. Regarding BTV several authors reported unexpected and inconclusive direct horizontal transmission with different serotypes (BTV8, BTV1, and BTV26 at least) [58,59,60,61].

Several authors reported a direct link between the inoculated viral doses and the onset of clinical signs and viraemia, i.e., the higher the dose the sooner the clinical signs and viral RNA detection [62,63,64]. In another study, we evaluated four 10-fold dilutions of a SBV infectious serum inoculum in ewes [30]. The undiluted original inoculum had a titre of 2 × 10^3^ TCID_50_/mL. It appears there is a critical dose to be inoculated for reproducing field-like virological and immunological parameters, and once this threshold is reached, there is no dose-dependent effect anymore. In the successfully infected animals, no statistical differences between the different inoculation doses were found in the duration or quantity of viral RNA circulating in blood, nor in the amount of viral RNA present in virus positive lymphoid organs. Likewise Di Gialleonardo et al. compared three groups of cattle inoculated with 100-fold dilutions of BTV8; no significant differences in viraemia kinetics could be found [65].

Inoculation by the bite of *Culicoides* was reported to be more efficient than intradermal inoculation, especially by delaying the early immune response of the host despite a generally lower inoculated viral dose when compared to needle inoculation [66]. Several mechanisms were hypothesized to explain this apparently enhanced infectivity in *Culicoides* transmitted BTV:

(1) The Culicoides saliva contains proteases able to cleave VP2, leading to the formation of infectious subviral particles (ISVP) displaying higher infectivity in KC cells and Culicoides [43];
(2) The ratio of infectious BTV particles versus defective virions produced within Culicoides might be higher when compared with cell culture grown BTV [66]; and
(3) Pharmacological agents contained in Culicoides saliva might affect the host’s immune response by anti-proliferative effects on leucocytes [67] or a reduced INF alpha/beta expression, as demonstrated with vesicular stomatitis virus and mosquito saliva [68].

Nonetheless, the use of *Culicoides* to perform experimental challenges remains highly limited by practical constraints: to date besides *C. nubeculosus, C. riethi*, and *C. sonorensis* no other *Culicoides* species were successfully establish as lab-adapted colonies [69,70], the alternative being insects caught in the wild. In addition, prior to the infectious challenge on the ruminant host, the infection of *Culicoides* is particularly tricky given the size of the insect and the exact amount of virus delivered to each ruminant cannot be known.

Altogether, the subcutaneous route seems to represent the best compromise for BTV and SBV. The dose itself has to be sufficient but there is no gain in using massive viral load.

#### 2.1.3. Screening for Concomitant Pathogens

Bluetongue disease history is scarred with incidents of contamination of biological samples. In 1992, modified live vaccines against canine distemper, canine adenovirus type 2, canine parainfluenza, and canine parvovirus, reconstituted with a killed canine coronavirus vaccine, led to abortions in several bitches. A virus could be isolated and was eventually identified as BTV serotype 11 [71,72]. More recently, a case of BTV11 contamination was reported by ANSES (*Agence nationale de sécurité sanitaire* *de l’alimentation, de l’environnement et du travail*, Maison-Alfort, France), in the context of an experimental infection of goats with BTV8. It appeared to be very closely related to the BTV11 isolated in Belgium [73]. We discussed a BTV15 contamination in a recent study [17]. This particular inoculum has been previously involved in two other experimental infections. Eschbaumer et al. used BTV1 culture supernatant that was then passaged once on VERO cells before being injected in calves and sheep [74]. That inoculum has been subsequently used by Dal Pozzo et al. [34], with the exact same outcome, namely discovery of the BTV15 contamination. BTV inoculums were not only contaminated with BTV heterologous serotypes: Rasmussen et al. reported the use of a BTV2 inoculum contaminated with Border Disease Virus in sheep [75].

So far, literature does not report experimental infections with a SBV inoculum that was contaminated by another virus belonging to the same or a different family. Broadly speaking contamination routes are most likely related to i) laboratory contamination during sample preparation or ii) natural multiple infection of the original donor animal [76]. Given the potential dramatic consequences of such contamination incidents, inocula should be tested for major pathogens affecting the host species used in challenge experiments but also for a set of BTV serotypes considered to be the most at risk. Despite the transient circulation of BTV6 [77], BTV11 [76], and BTV14 [78] of vaccine origin in Europe, the BTV11 contamination here above mentioned happened to be similar to BTV11 reference strain. Hence, the contamination of the inoculum is far from being necessarily related to an ongoing viral circulation even though it might remain silent because of the lack of clinical consequences. Thus, to rule out any potential BTV contamination all known BTV serotypes should be tested for. Such a recommendation would inevitably increase the constraints and costs of quality control of inocula prior to their use in experimental infections. Extensive screening could however be considered on a case-by-case basis.

## 3. BTV and SBV Display Placental Crossing Abilities and Teratogenic Potential

Vertical transmission from pregnant dams to their offspring is one of the major consequences for both SBV and BTV. Many pathogens are able of crossing the placenta to cause foetal injury. Most maternal virus infections are not transmitted to the foetus. However, certain viruses are able of crossing the placental barrier possibly causing developmental defects (teratogenesis). The teratogenesis is the production of a permanent abnormality in structure or function, restriction of growth, or death of the embryo or foetus [79]. The outcome of in utero infection depends on the susceptibility of the foetus to the infecting virus, which in turn, is a reflection of the gestational age of the foetus at exposure as well as the virulence characteristics of the infecting virus [80]. Nervous tissues are important targets for both BTV and SBV: usually the younger the foetus, the more severe the lesions [81,82].

To colonize the foetus viruses need a way in. Therefore, it is considered that SBV in utero infection can only occur once the first placentomes were established, around day 30 of pregnancy in cattle and slightly earlier in sheep [83,84,85]. At implantation, several changes occur: the papillae in the uterine glands immobilize the conceptus and it starts to elongate (cattle: 15 days post coitum (dpc); sheep: 13-16 dpc). Subsequently the cells of the trophectoderm and the uterine epithelium get interdigitated and binucleate cells start to be seen. Then binucleate cells start to differentiate and to migrate (cattle: 20-22 dpc; sheep: 16–18 dpc). Foetal villi develop in the caruncular areas starting at 24–26 dpc in small ruminants and 28–30 dpc in cattle, thus defining the end of the implantation and the start of the placental development [86]. Table 2 summarizes some of the essential events in the course of the prenatal development in cattle and sheep.

**Figure 1:**
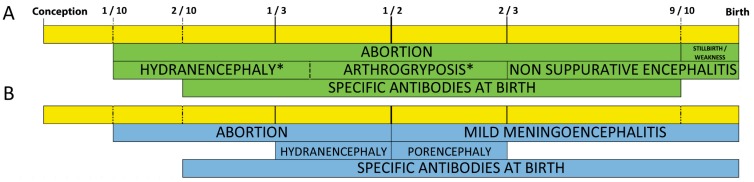
Schematic suggested timeframe for SBV (**A**) and BTV (**B**) in utero infection causing defects in cattle and small ruminants’ offspring [27,32,81,85,104,127,128]. In utero potential BTV (blue shades) and SBV induced defects (green shades) following infection of the pregnant dams along the whole gestation time for cattle and small ruminants. Time is expressed as a fraction of the total gestational time. A more detailed timeline is available as supplementary file.

In a recent study, we decided to infect with BTV8 vaccinated and non-vaccinated pregnant heifers at 120 days of pregnancy (BTV pregnant heifers study [32]). We also challenged pregnant ewes with SBV at 45 and 60 days of pregnancy (SBV pregnant ewes study [27]). Thus, for both viruses the experimental infection took place within the critical timeframe, between 30 and 150 days for cattle and between 30 and 70 days of pregnancy for sheep (Figure 1, [85]). Moreover, in experimental conditions the most frequent BTV transplacental infection was reported to occur at mid-term gestation, around 70 days of pregnancy in sheep [58,59].

The prenatal period can be divided into four main periods: i) fertilization; ii) blastogenesis; iii) embryogenesis; and iv) foetogenesis [94]. The embryo develops tissues and organ structures from the three original germ layers (ecto-, meso-, and endoderm). Once the organs are differentiated the embryo becomes a foetus [95]. The foetal phase is characterized by a fast growth of the conceptus. In cattle and sheep the foetogenesis starts around 45 and 38 dpc, respectively [90]. Thus, the critical timeframe for BTV and SBV infection overlaps the end of the embryo stage and the beginning of the foetal stage. Moreover, although in ruminants gamma globulins are unable to go through the placental barrier from the mother to the foetus it is admitted that cow and sheep foetuses become sequentially and increasingly immunocompetent to a larger variety of antigens throughout the pregnancy [96,97]. The critical timeframe for BTV and SBV infection also spans over the course of several important events during the immune system development (Table 2). Although the sequence of antigens to be successively and progressively recognized by the foetal ruminant through pregnancy seems to be quite conserved between individuals, these antigens can be recognized starting with a difference of a few days between individuals [98]. This individual variability could explain the findings by De Clercq et al. (2008), who reported all possible combinations of serological status/RTqPCR results in dam/calf pairs in a context of high BTV8 suspicion along with results which were interpreted as apparent immunotolerance [99]. Likewise, malformed calves and lambs were reported to be SBV viropositive or vironegative with or without SBV antibodies, suggesting the possibility of an in utero clearance of the virus. Moreover, most of the malformed calves that were negative in both SBV antibodies and RTqPCR were born from seropositive mothers [100].

In our studies, none of these evocative lesions was reported either in the BTV pregnant heifers study or in the SBV pregnant ewes study [27,32].

Following the infection of pregnant dams we reported the reddening of the muzzle and haemorrhages in the wall of the pulmonary artery in calves born from non-vaccinated mothers. Haemorrhages of the pulmonary artery is a BTV typical yet not pathognomonic lesion [101]. These findings were associated with the absence of any anti-BTV antibodies prior to the colostrum intake [32]. Melzi et al. reported the early infection and destruction of follicular dendritic cells following BTV infection. Consequently, antibody production is notably impaired and could be an element explaining the lack of BTV antibody detection in those calves [57]. This result provides an interesting perspective to the many petechial haemorrhages we observed on lymph nodes in several of our own experimental infections [14,17,32,33].

Following the infection of pregnant ewes with SBV, out of the 22 born-alive lambs none had any anti-SBV neutralizing antibodies prior to colostrum intake [27].

In both these experiments, timing of inoculation was optimal to achieve transplacental infection of the foetus with regard to data available from the literature yet no malformations could be seen. No antibodies against the virus used to infect the mothers could be detected as well. These striking results might even question the success of the infection, notwithstanding the positive RNA detection in the mothers. In our BTV pregnant heifers study, the report of similar lesions and serology results in another experiment on goats [102], and in our SBV pregnant ewes study the detection of SBV nucleic acids in organs of several lambs and many extraembryonic structures provided support to an actual transplacental infection. In addition, in another study [103] we managed to isolate SBV from foetal envelopes in the animals from the SBV pregnant ewes study at birth, thus 90 and 105 days post infection. The very low ratio of precolostral seroconversion in immunocompetent foetuses was also reported following the infection of pregnant cattle with SBV [104].

Transplacental transmission of BTV8 based on field data was reported to range from 16% [105,106] to 35% [107,108]. In experimental infections, passage of BTV8 from the mother to the foetus could be demonstrated in 43% of infected ewes whereas BTV1 could be detected in up to 67% of the foetus [59]. The highest susceptibility could be observed around 35-42 days of pregnancy in sheep [109] and infections after day 75 were reported to result in much lighter consequences [110]. Placental crossing, depending on the gestational stage, the BTV serotype and the inoculated dose, was reported to cause congenital defect in up to 40% of the offspring of infected ewes [111]. Other authors observed a BTV8 vertical transmission rate of 33% in goats infected at 61 days of pregnancy [112].

During the BTV epizootic of 2007–2008, Darpel et al., estimated transplacental infection rate of 33%, which is consistent with the latter result [107].

The lesions potentially presented by the calves affected in utero by SBV could be distinguished according to two entities: a hydrocephaly/hydranencephaly syndrome and a torticollis/arthrogryposis syndrome. By analogy with Akabane virus the infection during the first 6 months seems to be critical: an infection of the foetus between 76 and 104 days usually gives rise to hydranencephaly/porencephaly type lesions, and from 103 to 174 it is predominantly arthrogryposis [113]. The latest lesions have been observed for infection at 249 days of gestation and it appears that foetuses less than two months old (after conception) could be protected from in utero infection [113]. In contrast to SBV torticollis was hardly seen after a BTV8 in utero infection during the BTV epidemic in 2006–2009 but was more dominated by hydranencephaly in sheep [114].

Also in contrast to SBV, BTV in utero infection is in the vast majority of cases associated to BTV lab adapted strains (i.e., passaged on cell culture like modified live vaccine strains) or more recently with the European BTV8 wild type virus. By the end of the 20th century, at least five BTV serotypes of modified live vaccine origin (BTV4, BTV10, BTV11, BTV13, BTV17) were reported to be able to cross the placental barrier and possibly causing teratogenic effects [115,116,117,118]. In utero infection caused by wild-type strains was considered uncommon [119] yet documented [120].

SBV vertical transmission seems to be lower when compared to BTV, especially in cattle [121]. The rate of malformations caused by SBV was reported to be about 0.5% in cattle [122] although the rate of intrauterine infection—based on serological results of the calves prior to colostrum intake—was reported to be up to 28% [83]. Other authors documented field data about congenital malformations affecting 3% of the calves but 8-10% of the lambs in farms at the beginning of the SBV epizootic [123,124]. In Belgium based on a survey targeting farmers we also found an estimated 10% of malformed sheep in SBV positive flocks [125].

Table 3 summarizes the most common in utero malformations and nervous lesions induced by some of the most common viruses inducing BTV and SBV-like lesions in ruminants.

Embryonic losses represent a key factor affecting ruminant production systems. In cattle, as well as in sheep, most of the spontaneous embryo mortalities occur in the early embryonic life, namely before 16–18 dpc [129,130]. In cattle early embryonic losses under normal conditions were reported to range from 20 to 44% whereas in sheep in ranges from 12 to 30%, with a clear increase of embryo deaths with the ovulation rate [129,131]. The impact of both BTV and SBV on reproductive parameters other than teratogenesis is well documented. BTV8 was reported to increase the 56-days-return to service rate and the number of AI (Artificial Insemination) required to achieve pregnancy [132,133]. During the SBV epizootic the number of AI to get cattle pregnant was slightly yet significantly increased regardless of whether or not they were part of a herd reporting malformations indicative of an actual infection [134].

Although SBV and especially BTV had a tremendous economic impact on livestock industry, it is worthwhile highlighting the relative poor efficiency of the placental crossing and more specifically the overall low rate of congenital deformities induced by those viruses. However, congenital malformations underestimate the actual rates of BTV and SBV transplacental infections [128].

## 4. Conclusion and Future Prospects

The number of ruminants used in experimental infections is chosen based on welfare and statistical concerns but also quite unfortunately on economic and practical grounds [8]. We performed our experimental infections with BTV in the BSL3 facilities of Sciensano (Ukkel, Belgium) and with SBV in BSL2+/BSL3 facilities depending the phase of the experiment. Indeed, the Belgian Service of Biosafety and Biotechnology as well as the Belgian law classify BTV as a class 3 pathogen whereas there is no recommendation for SBV. Our biosafety measures for SBV were based on the analogy with AKAV, also classified as a class 3 pathogen [135]. Domestic ruminants being herd animals, need to be housed in groups or at least not individually. Euthanasia methods have to be the most humane as possible and clear end points have to be defined. Given the scarcity of clinical signs caused by BTV and SBV in the field and the individual variations in the response to the infection the number of animals to be included has to be chosen very carefully to comply with the Reduction objective (Three Rs concept) but has to be sufficient to limit the risk of not being able to provide useful data in the context of the ongoing scientific investigation. This is particularly difficult for experimental infection of pregnant ruminants with low malformation rates following transplacental transmission.

The most objective parameter to assess a vaccine efficacy against a virus and especially a RNA vector-borne virus is the evaluation of the viral RNA detection by RTqPCR in the host target [44]. BTV and SBV virulence was demonstrated to vary depending on the ruminant host whether it is cattle, sheep or goat. In addition, pregnancy length differs between cattle and small ruminants while the placentation and the development of the foetal envelopes present slight differences [136]. Consequently, to study any of the specific aspects related to a ruminant species there are no other animal models or any alternative able to mimic the natural situation in a proper way [8].

The results presented in our latest studies provide new insights in viruses that spread through Europe causing severe losses in livestock industry. In addition, these aspects open new perspectives to expand the knowledge on emerging vector-borne viruses targeting ruminants. More specifically, according to our experiments, the subcutaneous route with an inoculum passaged a limited number of times on cell culture seems to represent the best compromise between a high probability to reproduce an infection similar to what happens in the field and logistics concerning the preparation/storage/management of the inoculum. To prevent the loss of viral variability and limit the risks of attenuation, isolation of BTV could be done on KC cells [35], whereas SBV could benefit from an isolation on the highly susceptible SK-6 cell line [50]. Screening for concomitant pathogens should be considered on a case by case basis, if required. The dose should be chosen based on literature data yet no advantage is provided by inoculating a massive viral load.

Since some data from other authors suggest a better reproduction of the diseases with intradermal inoculation, it could be further investigated, especially if more user-friendly devices would be available. A major breakthrough would be the successful adaptation of a colony of Palearctic BTV and SBV vector *Culicoides* species (*C. obsoletus/scoticus, pulicaris*) to laboratory conditions and subsequent use in infectious challenges. Vector-borne transmission of BTV implies the puncture of the skin at some point. There is growing evidence that under certain circumstances additional routes of transmission can be observed: a goat was reported to be infected by BTV2 without direct contact [75]. The recently discovered BTV26 also displayed the ability to infect goats through direct contact [61]. In other experimental infections control ewes were found positive with BTV1 and BTV8 [58,59]. The study of the virus factors affecting this modified/underreported transmission feature should allow a better understanding of the epidemiology of the disease.

In conclusion, targeting ruminant host species in experimental infections especially with BSL3 *Culicoides* borne pathogens is very expensive, time consuming, subject to stringent animal welfare constraints and critical sample size analysis to meet optimal statistical requirements. However, ruminant model remains unavoidable to assess the disease impact and to study the pathogenesis of emerging vector-borne viruses.

## Figures and Tables

**Table 1:** Inocula characteristics used in the 10 most recent experimental infection studies on BTV and SBV (as searched on PubMed (July 2019) with keywords “experimental infection bluetongue” and “experimental infection Schmallenberg”). Only articles about experimental infection involving at least one ruminant species among cattle, sheep or goats were retained. The number of infected animals only takes into account actually infected ones, excluding control animals.

Virus	Type of Inoculum	No. and Species of Infected Animals	Cell Type	Number of Passages	Inoculation Route	Volume (mL)	Doses (TCID_50_/mL)	Reference
BTV8	Cell-passaged	16 sheep	KC	2	SC	1	10^5,75^	Flannery et al., 2019 [12]
BTV4 TV16	Cell-passaged	6 sheep	BHK21+KC	not specified	ID	1	10^6^	Putty et al., 2019 [13]
BTV1 BTV2 BTV4 BTV9 BTV16	Cell-passaged	30 cattle	BHK-21	2-4	SC	2,5-4	10^6^	Martinelle et al., 2018 [14]
BTV27	Cell-passaged blood (goat)	7 sheep, 13 goats and 4 cattle	BSR; ECE+BSR	3; 1+3 or 1+2	SC; IV (blood)	2, 3 or 4; 1 (blood)	10^3^–10^4.67^	Bréard et al., 2018 [10]
BTV4	Cell-passaged	4 sheep, 3 goats and 3 calves	KC+BHK-21	1+1	SC	2 – 4	10^6^	Schulz et al., 2018 [15]
BTV25 *	Reverse genetic	10 sheep and 2 goats	/	/	SC+IV	1	10^5^	van Rijn et al., 2016 [16]
BTV8	Cell-passaged	8 calves	BHK-21	2	SC+IV	1–4	10^4^–10^6.15^	Martinelle et al., 2016 [17]
BTV8	Cell-passaged	10 sheep and 4 cattle	KC	2	SC, ID	1	10^7^	Darpel et al., 2016 [18]
BTV8	Blood	8 sheep	/	/	ID	2	10^6.08^	Drolet et al., 2015 [19]
BTV8 BTV16	Cell-passaged	37 sheep	KC	3 and 2	SC	3	not possible	Bréard et al., 2015 [20]
SBV	Cell-passaged	13 cattle	BHK-21	4	SC	10	10^5^	Kęsik-Maliszewska et al., 2019 [21]
SBV	Serum (cattle)	35 cattle	/	/	SC	2 X 0.5	not specified	König P et al., 2019 [22]
SBV	Cell-passaged/sheep brain homogenate/serum (sheep)	10 sheep, 9 cattle	C6/36	1	SC	1 – 3	10^5.15^ and 10^3.15^**	Endalew et al., 2019 [23]
SBV	Serum (cattle)	25 goats	/	/	SC	1	/	Laloy et al., 2017 [24]
SBV	Plasma	9 sheep	/	/	IV	20	not specified	Rodríguez-Prieto et al., 2016 [25]
SBV	Serum (cattle)	5 sheep	/	/	SC	1	10^3.3^	Poskin et al., 2015 [26]
SBV	Serum (cattle)	17 sheep	/	/	SC	1	10^3.3^	Martinelle et al., 2015 [27]
SBV	Serum (cattle)	9 sheep	/	/	SC, ID, IN	1	10^3.3^	Martinelle et al., 2015 [28]
SBV	Serum (cattle), blood (sheep)	6 goats	/	/	SC	1	not specified	Laloy et al., 2015 [29]
SBV	Serum (cattle)	12 sheep	/	/	SC	1	10^3.3^, 10^2.3^, 10^1.3^ and 10^0.3^	Poskin et al., 2014 [30]

*: actually BTV1 and BTV6 expressing BTV25 proteins. **: converted in TCID50/mL from PFU using the formula PFU (mL)/TCID50 (mL) = 0.7 [31]. ID: intradermic; IN: intranasal; IV: intravenous; SC: subcutaneous.

**Table 2:** Key events in sheep and cattle embryos/foetuses with particular emphasis on nervous and immune systems. Compiled from [87,88,89,90,91,92,93].

Event	Timing in Cow (dpc)	Timing in Sheep (dpc)
Blastocyst hatching from zona pellucida	9	9
Elongation of the blastocyst, establishment of the primitive streak, emergence of the notochord	17–18	13–14
Appearance of neural folds, closure of the neural groove	17–19	15–16
Implantation begins	16–19	15–18
Neurula	20–21	17
Neural tube complete; optic and otic vesicles present	21–23	19–20
Placentation begins	22–23	17–22
Three brain vesicles visible	24–25	17
Placentoma are detectable	32–36	21
Lymphoid development of the thymus	42	36
Spleen development	55	43–44
Peripheral lymph nodes	60	45
IgM containing cells	59	65
Myelin sheath acquisition (starting)	60	54–63
IgG containing cells	145	87

With dpc, the days post coitum; IgM, immunoglobin M; IgG, immunoglobin G

**Table 3:** Summary of some of the most common central nervous and musculoskeletal lesions following in utero infection with bovine virus diarrhoea virus (BVDV), SBV, BTV, Akabane virus (AKAV), or Aino virus (AV). Adapted from [126].

Lesion	Definition	BVDV	SBV	BTV	AKAV/AV
Hydranencephaly	Extensive loss of cerebral tissue with replacement by clear fluid	+	+	+	+
Porencephaly	Cystic fluid filled cavities in the brain tissue	+	+	+	+
Hydrocephalus	Dilation of the lateral ventricles by cerebrospinal fluid	+	+	+	-
Microencephaly	Reduced size of the cerebrum	+	+	+	+
Cerebellar hypoplasia	Reduced size of the cerebellum	+	+	+	
Kyphosis	Dorsal vertebral column curvature	-	+	-	-
Lordosis	Ventral vertebral column curvature	-	+	-	-
Scoliosis	Lateral vertebral column curvature	-	+	-	-
Torticollis	Twisted cervical vertebral column curvature	-	+	-	-
Arthrogryposis	Joint contraction of the limbs	-	+	+/-	+

## Supplementary Materials

The following are available online at https://www.mdpi.com/1999-4915/11/8/753/s1, Figure S1: Schematic suggested timeframe for SBV (A) and BTV (B) in utero infection causing defects in cattle and small ruminants’ offspring [27,32,81,85,104,127,128].
