# Supplementary figures and images for: Reliable and Standardized Animal Models to Study the Pathogenesis of Bluetongue and Schmallenberg Viruses in Ruminant Natural Host Species with Special Emphasis on Placental Crossing
Source: Viruses. 2019 Aug 15;11(8):753. doi: 10.3390/v11080753 (PMC6722754; doi:10.3390/v11080753)

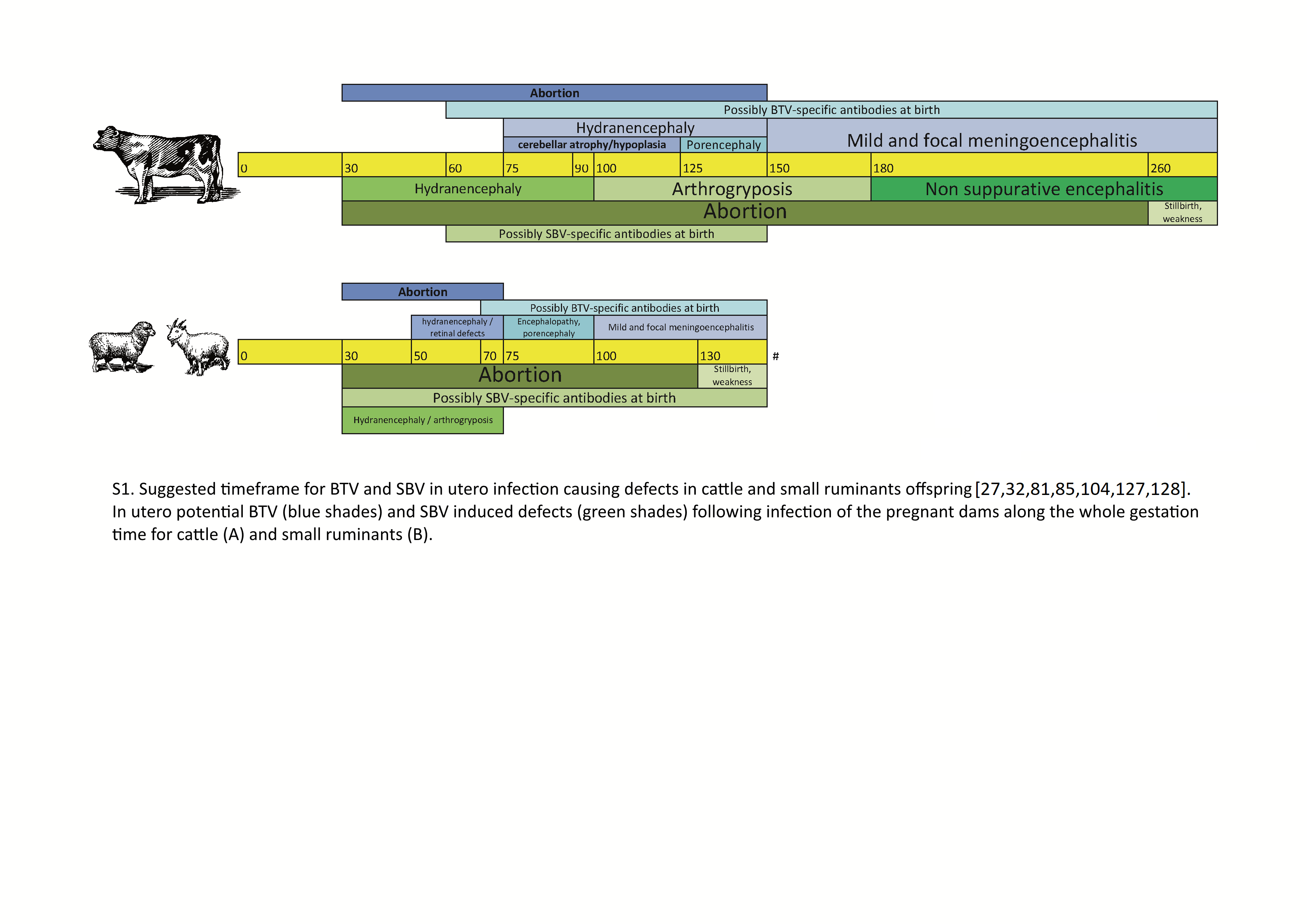

Supplement: Supplementary file 1 [file viruses-11-00753-s001.zip › viruses-525300-supplementary.tif]
